# Evaluation of molecular mouse sepsis panel: new portable and rapid tests for microorganism detection in suspected blood stream infection

**DOI:** 10.3389/fcimb.2025.1579074

**Published:** 2025-07-29

**Authors:** Claudia Colosimo, Maria Sofia Montanari, Anna Marzucco, Giulia Gatti, Lucrezia Puccini, Laura Grumiro, Laura Dionisi, Martina Brandolini, Ludovica Ingletto, Massimiliano Guerra, Giorgio Dirani, Silvia Zannoli, Alessandra Scagliarini, Alessandra Mistral De Pascali, Vittorio Sambri, Monica Cricca

**Affiliations:** ^1^ Unit of Microbiology, The Great Romagna Hub Laboratory, Pievesestina, Italy; ^2^ Department of Medical and Surgical Sciences (DIMEC), Alma Mater Studiorum-University of Bologna, Bologna, Italy

**Keywords:** molecular mouse, blood stream infection (BSI), molecular diagnoses, rapid test, sepsis

## Abstract

**Introduction:**

Bloodstream infections (BSIs) represent a significant public health concern, characterized by the presence of pathogens in the bloodstream, leading to serious conditions. Between 30 and 40% of BSIs evolve in sepsis, characterized by a life-threatening organ dysfunction and accompanied by a strong or dysregulated systemic immune or inflammatory response. A timely and accurate diagnosis of BSIs is crucial for ensuring effective management and treatment of critically ill patients. The diagnosis of BSIs can be initiated either from positive blood culture (BC) or directly from blood specimens using advanced diagnostic methods, these approaches are increasingly integrated into traditional diagnostic workflows.

**Methods:**

In the context of emerging molecular methods, Alifax introduced the Molecular Mouse (MM) Sepsis Panel in 2022. This study aims to evaluate the performance of MM cartridges in detecting Gram-positive bacteria and Gram-negative bacteria, as well as their associated resistance genes, using clinical leftover positive BCs and contrived samples for low-prevalence targets. Positive BCs were collected at the Unit of Microbiology, The Great Romagna Hub Laboratory, Pievesestina, FC, Italy.

**Results:**

Overall, MM Sepsis Panel showed excellent sensitivity (98.62%) and specificity (99.81%), confirming the system’s reliability and accuracy.

**Discussion:**

The high diagnostic accuracy demonstrated by these tests supports their potential application as rapid diagnostic tools for blood cultures, facilitating prompt identification of pathogens and antimicrobial resistance determinants in clinical microbiology settings.

## 1 Introduction

Bloodstream infections (BSIs) are a significant healthcare challenge, defined by the presence of pathogenic microorganisms in the bloodstream. These infections require prompt and accurate diagnosis to ensure effective treatment and improved patient outcomes (Kang et al., 2024). The clinical importance of BSIs stems from their rapid progression and the potential for systemic complications, necessitating prompt diagnosis and treatment (Arora et al., 2023). The incidence of BSIs has increased over time and reported BSI rates range from 122 to 220 cases/100,000 population. Rising incidence is probably related to an aging population and an increasing prevalence of underlying conditions and invasive procedures (Kontula et al., 2021). Infections that can cause sepsis are due to Gram-positive and Gram-negative bacteria, as well as fungal pathogens (Minasyan, 2017). In healthcare settings, multidrug-resistant organisms such as methicillin-resistant *Staphylococcus aureus* (MRSA) and carbapenem-resistant Enterobacteriaceae (CRE) are increasingly prevalent and pose significant therapeutic challenges (van Duin and Paterson, 2016; European Centre for Disease Prevention and Control, 2023). An estimated 47–50 million cases of sepsis occur worldwide each year, 80% of them are community-based, with children under the age of 5 involved in 40% of the cases (The 2030 World Sepsis Declaration, 2020). Worldwide, 1 in 5 deaths is associated with sepsis, accounting for at least 11 million deaths each year. In Europe, there are approximately 700,000 cases of sepsis annually. In Italy, the sepsis mortality rate has increased from 3% to 8% of all deaths in recent years (The 2030 World Sepsis Declaration, 2020). BCs are essential for detecting microorganisms in the bloodstream and for the timely diagnosis of bloodstream infections. Traditionally, the process involves incubation, Gram staining, and pathogen identification using MALDI-TOF MS (Idelevich et al., 2014). Automated antibiotic susceptibility tests typically provide results within 8 to 18 hours (Vandepitte et al., 2003; Lamy et al., 2020). In recent years, new technologies—encompassing both phenotypic and genotypic tests—have emerged that significantly reduce turnaround times (Peker et al., 2018; Antibiotic Use in the United States, 2021). Rapid diagnostic tools are pivotal in mitigating the overuse of broad-spectrum antibiotics and in addressing the escalating threat of antibiotic resistance on a global scale (Vandepitte et al., 2003). These tools enhance diagnostic efficiency and support faster, more targeted treatment of severe infections (Cherkaoui et al., 2021; Tibbetts et al., 2022; Esse et al., 2023). Innovative phenotypic systems such Radian^®^ by Copan Diagnostics (Italy), VITEK^®^ REVEAL™ by bioMérieux (France), and ASTar^®^ by Q-linea AB (Sweden) offer semi-automated tests for early detection of antibiotic resistance in positive blood cultures. Among genotypic tests, the FilmArray^®^ Blood Culture Identification (BCID) Panel (bioMérieux), Xpert^®^ MRSA/SA BC (Cepheid) enable rapid identification of pathogens and antibiotic resistance genes from positive blood cultures. while the T2Bacteria^®^ Panel (T2 Biosystems) detects pathogens directly from whole blood specimens. In this context, Alifax has developed the Molecular Mouse (MM) platform and 5 sepsis panels, each with different specificities (Mauri et al., 2024). The 5 Sepsis Panel cartridges allow the identification of various pathogens, gram-negative and gram-positive bacteria as well as candida species and their associated resistance genes. This study aimed to evaluate the performances of the bacterial MM sepsis panel cartridges compared to standard methods used in routine practice at the Unit of Microbiology, The Great Romagna Hub Laboratory, Pievesestina, FC, Italy.

## 2 Materials and methods

### 2.1 Sample description and identification

A total of 1583 positive BC specimens were analyzed in this study including both clinical leftover specimens and contrived specimens. Given the presence of polymicrobial specimens containing more than one pathogen, the resulting number of microbial isolates tested in this study was 1675. From December 2021 to May 2022, a total of 1458 leftover positive BC were collected at the Unit of Microbiology, The Great Romagna Hub Laboratory, Pievesestina, FC, Italy. All samples were anonymized before being included in the study (protocol code AVR-PPC P09, rev. 2, based on (Burnett et al., 2007), in line with the regulations issued by the Romagna Local Health Authority Ethical Board. Clinical leftover specimens were characterized as part of routine laboratory analysis for species identification via MALDI-TOF mass spectrometry (Vitek MS, bioMérieux) and Antimicrobial Susceptibility Test (AST), via VITEK 2 system (bioMérieux). The Vitek MS (bioMérieux) based on spectrometry MALDI-TOF use the colony picked and smeared on a disposable target slide (VITEK MS-DS TARGET SLIDE, (bioMérieux) and covered directly with 1μl of the matrix solution (VITEK MS-CHCA MATRIX, bioMérieux). The Antimicrobial Susceptibility Test (AST) was performed using VITEK 2 instrument (bioMérieux) using the following VITEK 2 cards: ST-N659 for *Staphylococci* spp., VITEK 2 AST-N658 for *Enterococci* spp., for VITEK 2 AST-ST03 for *Streptococci* spp., VITEK 2 AST-N376 for gram negative species and VITEK 2 AST-N397 for non-fermenting bacteria and multidrug resistant bacteria. The results were interpreted by the Advanced Expert System™ (bioMérieux), according to the EUCAST breakpoints and following the EUCAST recommendations versions 11.0 and 12.0, valid from 1 January 2021 to 31 December 2022. In routine diagnostics, in cases of phenotypic resistance or intermediate susceptibility to carbapenems in Enterobacterales, to methicillin in *Staphylococcus aureus*, and to vancomycin in *Enterococcus* spp., molecular analyses were additionally performed using GeneXpert^®^ panels (Cepheid, Sunnyvale, California, USA) to determine the resistance gene profile: Xpert^®^ Carba-R for the detection of KPC, NDM, VIM, IMP, and OXA-48; Xpert^®^ MRSA/SA for the detection of *Staphylococcus aureus* and methicillin resistance; and Xpert^®^ vanA/vanB for the detection of vancomycin resistance genes in *Enterococcus* spp (**Table 1**). Contrived specimens were employed to assess low-prevalence pathogens and resistance genes. A total of 125 BCs were prepared by inoculating well-characterized or reference strains from different culture collections (ATCC, American Type Culture Collection; NCTC, National Collection of Type Cultures; DSMZ, German Collection of Microorganisms and Cell Cultures GmbH; CCUG, Culture Collection University of Gothenburg) as follows: a volume of 2 mL of microbial suspension at a concentration of 100–200 CFU/mL were inoculated into a BACT/ALERT^®^ PLUS aerobic/anaerobic blood culture bottles (bioMérieux, Marcy l’Etoile, France) along with 8 mL of human whole blood for a total of 10 ml (in accordance with the manufacturer’s specifications). The contrived blood culture bottles were incubated in the BacT/AlertVirtuo^®^ instrument (bioMerieux) until flagged as positive by the automated culture system. See **Supplementary Material** for more details on the strains used for contrived specimen preparation (**Supplementary Table S1**). A detailed description of the tested samples for each MM cartridge is summarized in **Supplementary Material**.

**Table 1:** The table shows the association between the antibiotic tested, interpreted by the Advanced Expert System™ (bioMérieux), and the resistance genes that detect MM panels.

Microrganism	CARD VITEK 2	Breakpoints according to EUCAST v11 and v12)	Gene resistance on MM system
*Enterobacterales*	376/397	Colistin >2 mg/L	mcr-1
			mcr-2
*Enterobacterales*	376/397	Meropenem > 8 mg/L Ertapenem > 0.5 mg/L	OXA-23-like
			IMP
			KPC
			VIM
			OXA-48 like
			NDM
*Enterobacterales*	376/397	Cefotaxime >2 mg/L Ceftazidime >4 mg/L	CMY-2
			CTX-M-2/8 group
			CTX-M-1/9 group
			SHV
			SHV ESBL
*Enterococci*	658	Vancomycin >4 mg/L	vanB
			vanA
			vanC1
			van C2-3
*Stafilococci*	659	Positive Cefoxitin screen	mecA
			mecC
			SCCmec-orfX

### 2.2 Molecular mouse platform

The MM platform is a qualitative non-automated *in vitro* diagnostic device intended as an aid to the diagnosis of microorganisms in positive human blood culture from generic population. It consists of a compact thermocycler for sample amplification, a ready-to-use cartridges with 6 microwells containing all necessary freeze-dried reagents to perform the multiplex Real-Time PCR, and a closed software for data analysis and result management. The MM platform performs analysis of specific DNA target sequences by means of disposable lab-on-a-chip devices, based on Silicon technology. Specifically, 4 different cartridges were employed in this study: MM GRAM NEG RES (SI 1701.0101/L), MM GRAM NEG ID (SI 1701.0102/L), MM GRAM POS STAPH (SI 1701.0103/L), and MM GRAM POS NO STAPH (SI 1701.0104/L). These cartridges were utilized for the detection of nucleic acid sequences specific to Gram-negative and Gram-positive bacteria, as well as genetic markers associated with antimicrobial resistance, including those encoding for carbapenemases, extended-spectrum beta-lactamases (ESBL), AmpC-type enzymes, and resistance to colistin, vancomycin, and methicillin. A more detailed list of the MM cartridge targets is provided in the **Supplementary Material** (**Supplementary Table S2**).

### 2.3 Sample preparation for MM analysis

The MM platform (Alifax s.r.l) for the identification of bacterial species and their resistance genes starting from positive BC does not require a DNA extraction/purification step, but only a simple and fast pre-analytical procedure. In more detail, a 200 µL volume of positive BCs was centrifuged at 500g for 1 minute (min). The supernatant was then transferred in a new tube and centrifuged again at 5000g for 1 minute. The final supernatant was discarded, and the pellet was resuspended in 1 mL of molecular biology grade water. A dilution was then performed with the loading solution provided within the kit, following the manufacturer’s instructions. 5 µL of the solution were then added into each well of the cartridge to resuspend the freeze-dried reagents. The cartridges were loaded into the MM instrument and recognized automatically through the RFID tag. Once the analysis starts, the MM results are available in approximately 1 hour.

### 2.4 Study setting

**Figure 1:**
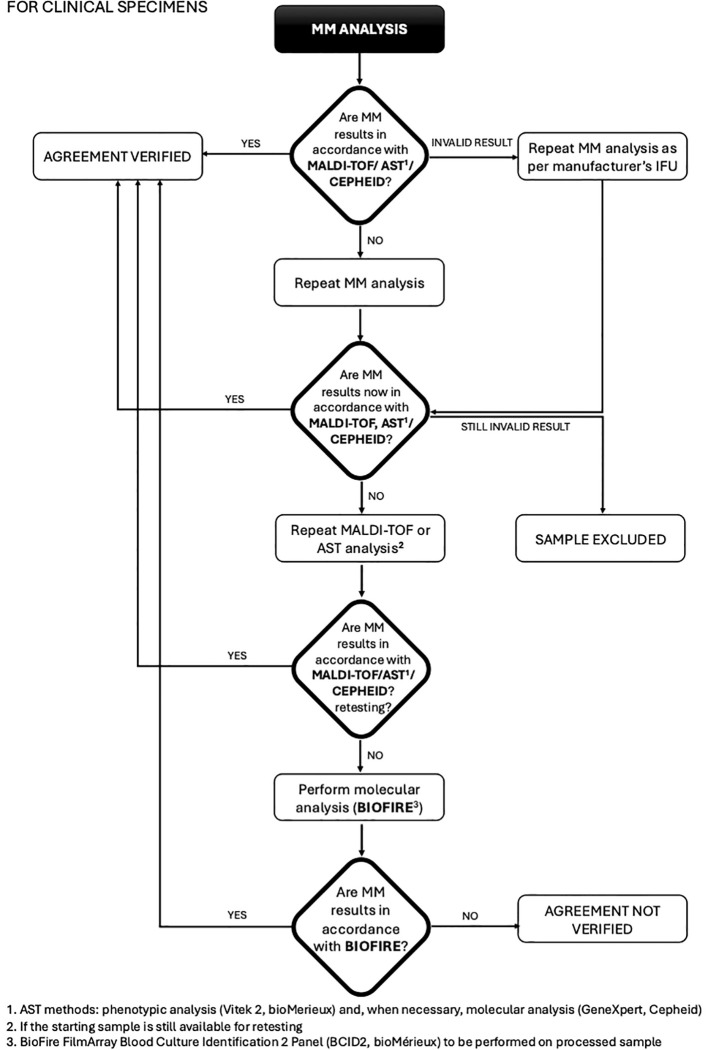
Flowchart for clinical specimens from blood colture: procedure and management of discrepancies (MALDI-TOF/AST/CEPHEID).

**Figure 2:**
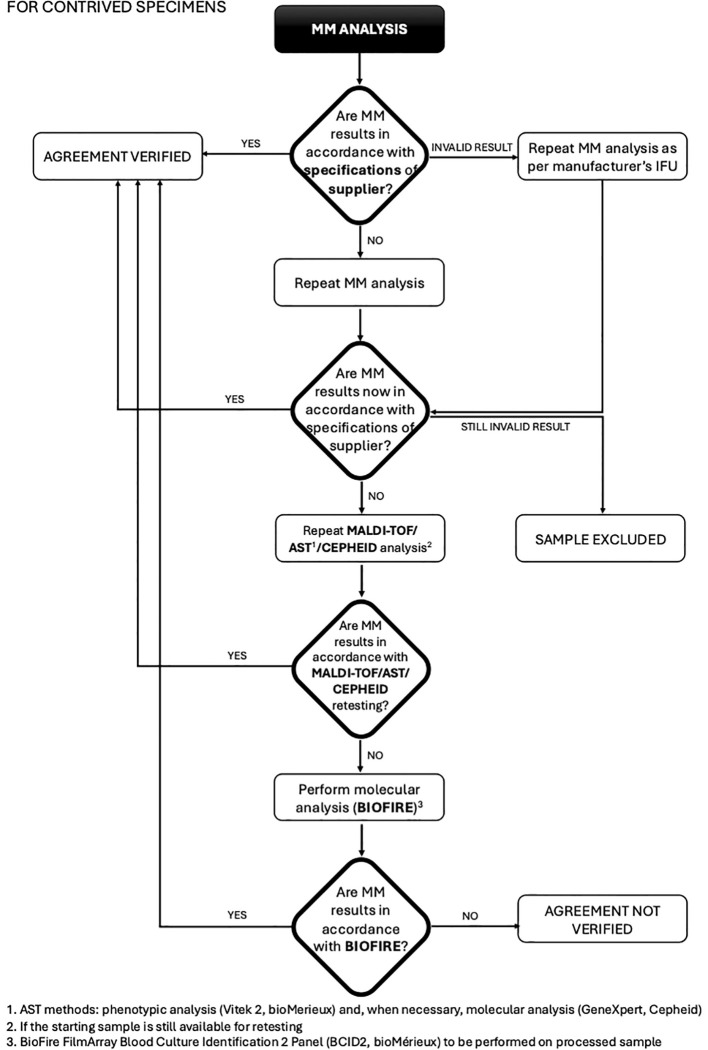
Flowchart for contrived specimens: procedure and management of discrepancies (MALDI-TOF/AST/CEPHEID).

This study was conducted at the Unit of Microbiology, The Great Romagna Hub Laboratory, Pievesestina, FC, Italy. MM analyses were performed according to the manufacturer’s instructions for use. In case of discrepancy between MM analysis and expected outcomes (outcomes derived from routine testing for leftover specimens or outcomes derived by pathogen specifications provided by the strain suppliers for contrived specimens), a test repetition was performed with a new dedicated MM cartridge. If the discrepancy persisted, MALDI-TOF or AST was re-performed to rule out any upstream contamination. If the disagreement remained unresolved, the sample was further analyzed using a reference molecular nucleic acid-based assay, the BioFire FilmArray Blood Culture Identification 2 Panel (BCID2, bioMérieux), and conclusions were drawn based on this secondary method. For invalid results due to a Positive Control “Not Pass”, caused by inhibitors in the specimen (Buckwalter et al., 2014; Sidstedt et al., 2020) re-analysis was performed according to the MM device instructions. Persistently invalid results led to sample exclusion. This approach ensured a rigorous evaluation of discrepancies and upheld the integrity of the diagnostic process. The study design was detailed and illustrated in a flowchart in **Figures 1**, **2**.

### 2.5 Data analysis

The sensitivity and specificity of the MM cartridges (Alifax s.r.l.) were assessed and calculated for each device analyte as follows: Positive Percent Agreement or Sensitivity (expressed in %) = 100 x TP/(TP+FN); Negative Percent Agreement or Specificity (expressed in %) = 100 x TN/(TN+FP) TP (true positive): the number of specimens with positive test result for which also the comparative method gave positive result; TN (true negative): the number of specimens with negative test result for which also the comparative method gave negative result; FP (false positive): the number of specimens with positive test result for which the comparative method gave negative result; FN (false negative): the number of specimens with negative test result for which the comparative method gave positive result. 95% confidence intervals for the observed agreement were calculated using the Wilson score interval method.

## 3 Results

A total of 1583 positive BC samples were analyzed in this study including both leftover positive BC specimens and contrived specimens.

### 3.1 Gram negative bacteria identification by MM GRAM NEG ID cartridge

**Figure 3:**
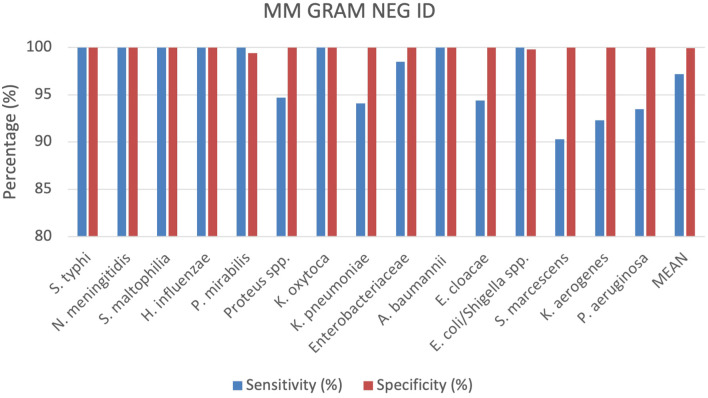
Graphical representation of the evaluation of the MM GRAM NEG ID cartridge results.

A total of 529 positive BC specimens including 165 leftover positive BCs prospectively collected, 158 leftover positive BCs retrospectively collected, and 206 contrived specimens were analyzed by the MM GRAM NEG ID cartridge (SI 1701.0102/L, Alifax s.r.l). A total of 18 polymicrobial BCs, all containing 2 different pathogen species, were also included. Sensitivity and specificity were calculated for each target included in the MM GRAM NEG ID cartridge (**Table 1**), 5 out of 15 targets analyzed showed 100% sensitivity and specificity. The sensitivity was found to be less than 100% for the following strains: *Proteus* spp. (94.7%), *K. pneumoniae* (94.1%), *Enterobacteriaceae* (98,5%), *E. cloacae* (94,4%), *S. marcescens* (90.3%), *K. aerogenes* (92.3%) and *P. aeruginosa* (93.5%). A total of six FN results were observed for generic targets: two for *Proteus* spp. and four for *Enterobacteriaceae*. Among the 38 samples positive for *Proteus* spp., 32 were identified as *P. mirabilis* and six as *P. vulgaris*. The two FN results for *Proteus* spp. corresponded to *P. mirabilis* that was successfully detected by the species-specific target in the MM system. Among the 270 bacteria belonging to the *Enterobacteriaceae* family identified by the reference culture-based method including 21 *Salmonella Typhi*, 22 *Klebsiella oxytoca*, 51 K*. pneumoniae*, 36 *Enterobacter cloacae*, 114 *Escherichia coli*, and 26 *Klebsiella aerogenes* a total of four FN results were recorded. Specifically, one FN sample corresponded to *E. cloacae*, one to *K. oxytoca*, and two to *K. pneumoniae*. The first two were detected through species-specific targets, while the remaining two were detected by nucleic acid-based testing via species-specific detection but were not detected by either the generic or species-specific targets in the MM system. Conversely, while three *K. pneumoniae*, two *E. cloacae*, and two *K. aerogenes* samples were not identified by species-specific targets, they were detected by the generic *Enterobacteriaceae* target, with the exception of one *K. pneumoniae* isolate, which was not detected by either target but was confirmed by the nucleic acid-based comparator method. Additionally, six FN results were associated with the species-specific targets: three for *Serratia marcescens* and three for *Pseudomonas aeruginosa.* The specificity was 100% for all targets except for *P. mirabilis* and *E. coli/Shigella* spp. whose specificities were 99.4% and 99.8%, respectively (**Figure 3**). One FP result was observed for the *E. coli/Shigella* spp. target out of total 414 negative samples. Among the three FP results for the *P. mirabilis* species-specific target, one was also detected by the generic target using both the MM system and the nucleic acid-based comparator method. More details can be found in the **Supplementary Materials** (**Supplementary Table S3**).

### 3.2 Gram negative bacteria resistance detection by MM GRAM NEG RES cartridge

**Figure 4:**
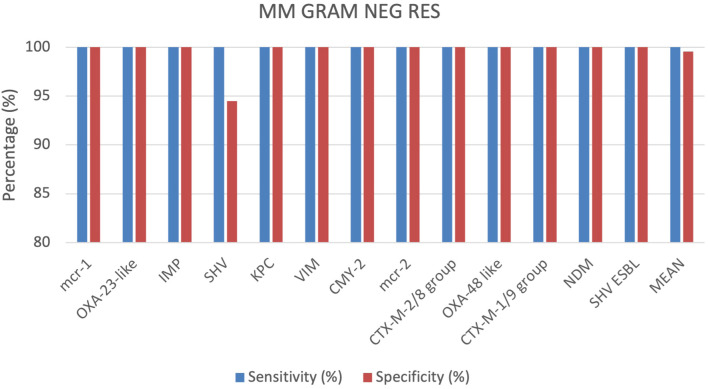
Graphical representation of the evaluation of the MM GRAM NEG RES cartridge results.

A total of 355 positive BC specimens, including 76 prospective leftover positive BCs samples, 61 retrospective leftover positive BCs samples and 218 contrived samples were analyzed using the MM GRAM NEG RES cartridge (SI 1701.0101/L, Alifax s.r.l). 4 out of 355 specimens were polymicrobial BCs, each containing 2 different pathogen species. Based on the outcomes achieved, sensitivity and specificity were calculated for each target included in the MM GRAM NEG RES cartridge. The sensitivity was 100% for all targets (**Figure 4**). The specificity was 100% for all targets, except for the SHV and OXA-48-like targets, which displayed sensitivity of 91.7% and 98.8%, respectively (**Figure 4**). In 21 of the 252 total samples that tested positive for the SHV target, the Gram-negative bacteria identified were susceptible to ceftazidime and cefotaxime according to AST results. Three FP results for the OXA-48-like target were observed when compared both to susceptible meropenem and ertapenem, and these findings were further confirmed by nucleic acid-based testing. More details can be found in the **Supplementary Materials** (**Supplementary Table S4**).

### 3.3 Staphylococci identification by MM GRAM POS STAPH cartridge

**Figure 5:**
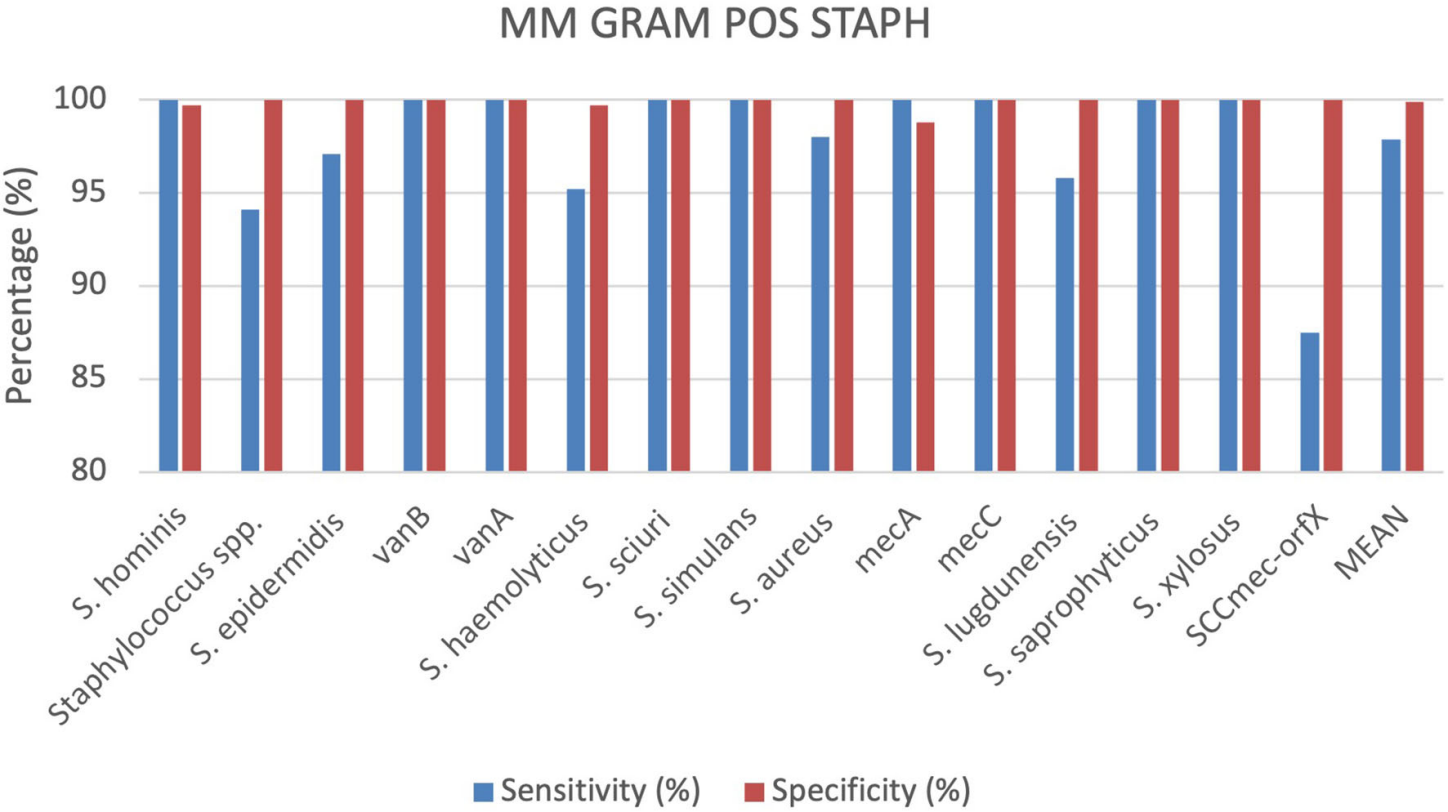
Graphical representation of the evaluation of the MM GRAM POS STAPH cartridge results.

A total of 336 positive BC specimens, including 134 prospective leftover positive BC samples, 94 retrospective samples and 108 contrived samples were analyzed using the MM GRAM POS STAPH cartridge (SI 1701.0103/L, Alifax s.r.l). 38 specimens were polymicrobial BCs, including 37 double species and 1 triple species. Sensitivity and specificity were calculated for each target included in the MM GRAM POS STAPH cartridge. The device displayed 100% sensitivity for nine targets, including *S. hominis, S. sciuri, S. simulans, S. saprophyticus, S. xylosus* and the vanB, vanA, mecA and mecC resistance genes. Sensitivity values of the remaining targets ranged from 87.5% to 98%. Among ten FN results for generic target *Staphylococcus* spp., eight were correctly detected by species-specific target (7 *S. sciuri* and 1 *S. simulans*) while the remaining two were *S. warneri* isolates. A single FN result was observed for each of the following targets: *S. epidermidis*, *S. haemolyticus*, *S. aureus* and *S. lugdunensis.* In the case of *S. epidermidis* and *S. haemolyticus isolates*, generic *Staphylococcus* spp. target was correctly detected along with *mecA* gene. Similarly, in the *S. aureus* FN case, the genus-level target, *mecA* and *SCCmec-orfX* were detected. Among the resistance genes targets included in the cartridge, all demonstrated 100% specificity and sensitivity, apart from the SCCmec-orfX gene cassette. Three FN results were observed for this target, as the reference method identified MRSA strains by cefoxitin screen (Vitek 2 AST-659) and Cepheid MRSA Blood cartridge. Specifically, in one case, the MM system detected *Staphylococcus* spp., *S. aureus*, and *mecA*. In the other two cases, the analytes detected were *Staphylococcus* spp., *S. aureus*, and *mecC*, but no amplification was observed for SCCmec-orfX. Specificity was 100% for all targets except *S. hominis* (99.7%), *S. haemolyticus* (99.7%) and mecA (98,8%) (**Figure 5**). *S. hominis* was detected along with MRSA in one sample but could not be evaluated by the nucleic acid-based comparator due to the lack of the corresponding target in its detection panel. One FP result was observed for *S. haemolyticus*, which was identified as *S. epidermidis* by the culture-based reference method. The discrepancy was further evaluated using a nucleic acid-based comparator method, which lacks a specific target for *S. haemolyticus*. This method did not detect *S. epidermidis*, but identified only the genus-level target (*Staphylococcus* spp.). In one sample, culture-based reference method identified a single pathogen, *Streptococcus anginosus*, whereas the MM system detected *Staphylococcus* spp. and *mecA* resistance gene. This discordance was assessed using the nucleic acid-based comparator method, which detected both *S. anginosus* and *Staphylococcus* spp., but could not rule out the presence of *mecA* gene due to limited detection algorithm. More details can be found in the **Supplementary Materials** (**Supplementary Table S5**).

### 3.4 Gram positive bacteria No Staphylococci identification by MM GRAM POS NO STAPH cartridge

**Figure 6:**
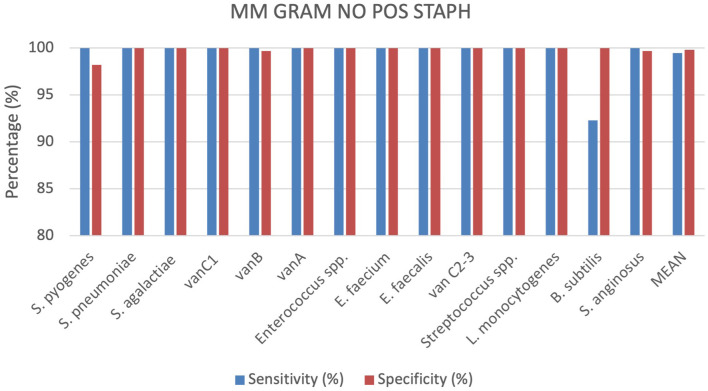
Graphical representation of the evaluation of the MM GRAM NO STAPH cartridge results.

A total of 363 positive BC specimens, including 148 leftover positive BCs prospectively collected, 99 retrospectively collected and 116 contrived samples, were analyzed by the MM GRAM POS NO STAPH (SI 1701.0104/L, Alifax s.r.l). 29 polymicrobial BCs, including 28 double species and 1 triple species, were tested. Sensitivity and specificity were calculated for each target included in the MM GRAM POS NO STAPH cartridge, 5 out of 15 targets analyzed showed 100% sensitivity and specificity. The device demonstrated 100% sensitivity for all targets, with the exception of *B. subtilis* (92.3%) for which one FP result was observed. Specificity reached 100% for all targets, except for *S. pyogenes, vanB* and *S. anginosus* which demonstrated specificity of 98,2%, 99.7% and 99.7%, respectively (**Figure 6**). Four *Streptococcus dysgalactiae subsp. dysgalactiae* and two *Streptococcus mitis* isolates were misidentified as *S. pyogenes* but were correctly assigned to the genus *Streptococcus.* In one sample, culture-based reference method identified a single pathogen, *S. pyogenes*, whereas the MM system additionally detected *S. anginosus* alongside this isolate. This discordance was not assessed using the nucleic acid-based comparator method, which does not include *S. anginosus* in its detection panel. 96 samples containing *Streptococcus* spp. were tested and correctly detected by the generic target, including 20 *S. pneumoniae*, 18 *S. pyogenes*, 19 *S. agalactiae*, 20 *S. anginosus* and 19 other *Streptoccoccus* spp. The Xpert^®^ vanA/vanB cartridge identified the *vanA* gene in one vancomycin resistant *E. faecium* isolate, while the MM system detected both *vanA* and *vanB* resistance genes. More details can be found in the **Supplementary Materials** (**Supplementary Table S6**).

### 3.5 Evaluation of all cartridges analysed.

**Figure 7:**
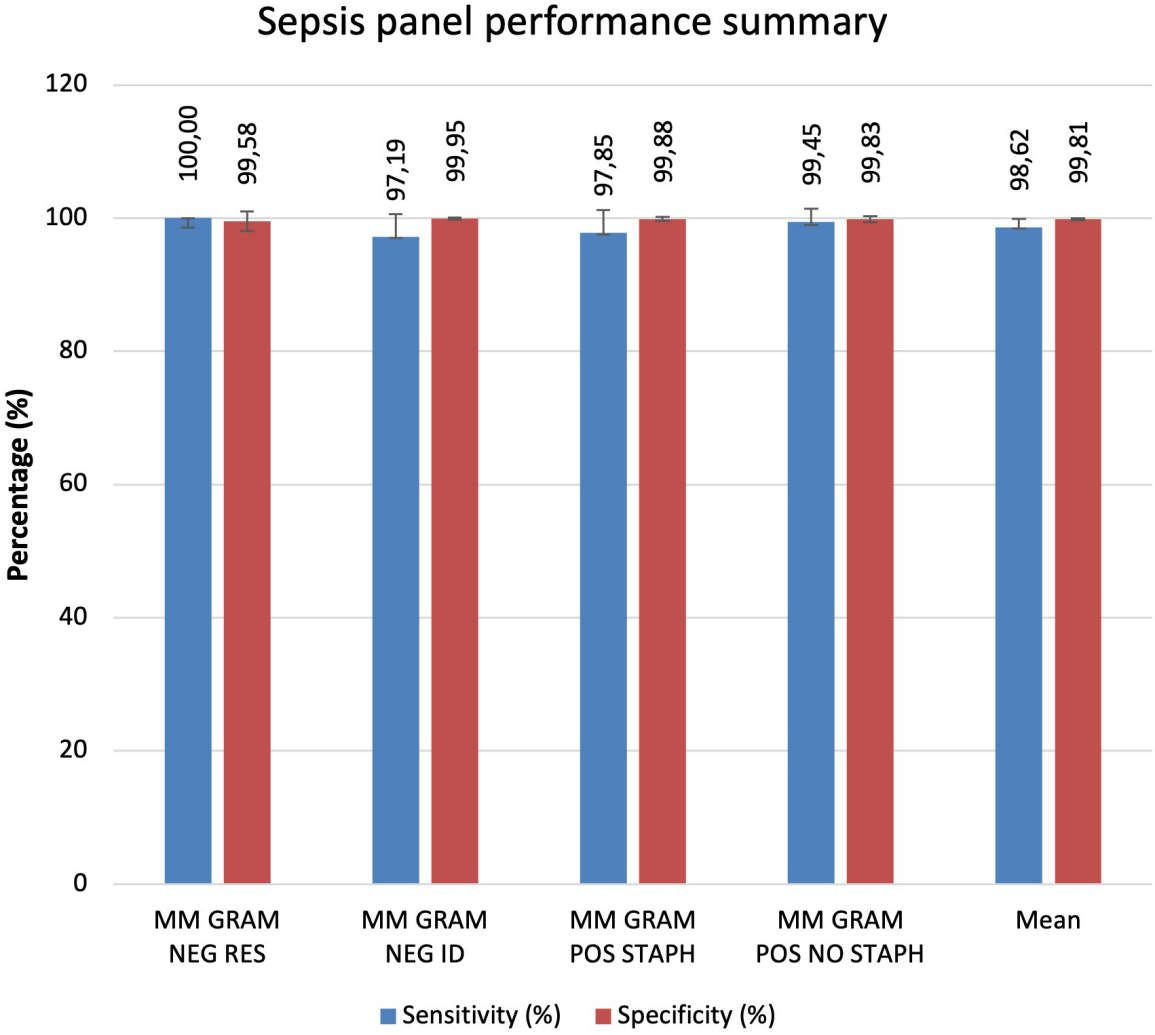
Graphical representation of the results of the evaluation of all cartridges analysed.

Comparing all cartridges used the results show that the sensitivity and specificity are both very high for all analysed cartridges. On average, specificity is slightly higher at 99.81%, compared to sensitivity at 98.62%. The MM GRAM NEG ID and MM GRAM POS STAPH cartridges show slightly lower sensitivity than specificity. The MM GRAM NEG RES cartridge reports 100% sensitivity. In the **Figure 1** a graphical representation of the results of the evaluation of all cartridges analysed (**Figure 7**).

## 5 Discussion

The timely identification of pathogens in BSIs remains a critical challenge in sepsis management, as delayed diagnosis can impede appropriate treatment of patients (Idelevich et al., 2014). Molecular diagnostic platforms have emerged as effective complement to traditional culture-based methods, offering significantly reduced turnaround times for both pathogen identification and resistance gene detection and enabling a prompt and appropriate treatment (Land et al., 2019). This study assessed the diagnostic performance of four MM cartridges targeting Gram-positive and Gram-negative bacteria, as well as their key antibiotic resistance genes. Overall, the MM system demonstrated high sensitivity and specificity across most targets, making it a reliable tool for rapid microbial diagnosis. MM GRAM NEG RES cartridges showed 100% sensitivity and specificity for all resistance markers, except for SHV and OXA-48-like genes. The SHV specificity was slightly lower (91.7%) due to detection of plasmid-borne SHV isoforms, widely disseminated or even endemic (Liakopoulos et al., 2016), with limited clinical significance. OXA-48-like specificity was 98.8%, with false positives likely due to sample contamination. On the other hand, MM GRAM NEG ID cartridges had an overall sensitivity of 97.2% and specificity of 99.9%. Reduced sensitivity was noted for *Proteus* spp. (94,7%) and *Enterobacteriaceae* (98,5%), due to targets’ inability to detect certain isolates for sequence mismatches (as declared in manufacturer’s instructions for use); however, this lower sensitivity for identifying family- or genus-specific targets has in some cases been mitigated by the detection of the species-specific targets, thus allowing for accurate detection. A lower sensitivity was also observed for *K. pneumoniae* and *E. cloacae*, largely due to limitations in primer design that targeted only species-specific sequences rather than groups or complexes. False positives were few and largely attributed to non-specific amplification artifacts. MM GRAM POS STAPH cartridge showed an average sensitivity of 97.8% and specificity of 99.9%. The sensitivity dropped for *Staphylococcus* spp. (94.1%), due to the lack of detection of *S. simulans*, *S. sciuri*, and *S. warneri* strains, mainly attributed to sequence mismatches in binding regions, in line with the *in silico* prediction (as declared in manufacturer’s instructions for use). Despite these issues, diagnostic accuracy for most cases was preserved thorough species-specific targets. Importantly, SCCmec-OrfX, specific for the detection of Methicillin-resistant *Staphylococcus aureus* (MRSA), showed an 87.5% sensitivity due to its capability to detect 11 out of the 14 described SCCmec types (Uehara, 2022), as predicted by *in silico* analysis; mecA amplification allowed accurate MRSA identification in discrepant samples. Lastly, the MM GRAM POS NO STAPH exhibited 100% overall sensitivity and specificity, with few exceptions: lower sensitivity for *Bacillus subtilis* (92.3%) due to weak amplification signals and lower specificity for *S. pyogenes* (98,2%), vanB (99.7%) and *S. anginosus* (99.7%), rather caused by target misclassification. The MM platform combines speed, modularity, and ease of use, delivering actionable results in approximately one hour starting from positive BC. Although it is not currently classified as a point-of-care test (POCT), its compact design and user-friendly interface make it suitable for decentralized diagnostics and integration into clinical microbiology workflows. An important practical feature is its cartridge-based modularity, which allows concurrent processing of different tests (e.g., Gram-positive ID, resistance, or mixed samples) using a shared software interface. The need for a pre-analytical Gram stain to select the appropriate cartridge is both a limitation and a cost-containment advantage, enabling tailored testing based on the microbial profile. Additionally, MM detects a broad spectrum of resistance determinants beyond the capabilities of the main reference methods (FilmArray BCID2, BioFire, bioMérieux), such as (i) OXA-23, important for carbapenem resistance in *Acinetobacter baumannii*, a critical pathogen as reported by WHO (World Health Organization) (WHO Bacterial Priority Pathogens List, 2024) (ii) vanC1/C2/3, which detect low-level vancomycin resistance in Enterococci (Courvalin, 2006); (iii) CMY-2 and SHV-ESBL, which improve the understanding of β-lactam resistance epidemiology (The EUCAST guideline on detection of resistance mechanisms v 2.0, 2017). These features allow the system to play a dual role in clinical decision-making and antimicrobial stewardship, supporting both individual patient care and institutional infection control. Despite promising results, several limitations of this study must be acknowledged. Firstly, only four of the available MM cartridges were evaluated. The MM YEAST BLOOD cartridge, relevant for fungal BSIs, was not included and should be considered in future evaluations to fully validate the system’s coverage. Secondly, many of the resistance targets, particularly in the MM GRAM NEG RES cartridge, were tested on simulated samples or certified strains (**Supplementary Material S6**). While suitable for analytical sensitivity assessments, these samples may not fully reflect the diversity and complexity of real-world clinical isolates. Moreover, the limited number of samples for low-prevalence pathogens or resistance genes led to wide confidence intervals. Also, some false positives that resulted from non-sigmoidal amplification curves were misinterpreted by the software as positive. While rare, these events suggest that improvement of algorithmic thresholds or manual curve validation could further improve result reliability. Furthermore, sensitivity limitations related to primer design were noted for specific target such as *E. cloacae* and *K. pneumoniae* for MM GRAM NEG ID and SCCmec-OrfX for MM GRAM POS STAPH. These gaps highlight the need for continuous primer optimization to accommodate genomic variability across strains and species. Another limit of the study is that performance for the detection of certain resistance gene targets was evaluated only through a comparison with the phenotypic results. Nevertheless, specific resistance phenotypes often correlate strongly with known resistance genes. For example, resistance to third-generation cephalosporins in Enterobacterales (e.g., cefotaxime or ceftazidime) often suggests the presence of ESBLs such as *bla*CTX-M, whereas carbapenem resistance in *Acinetobacter baumannii* may point to the presence of carbapenemase genes such as *bla*OXA-23*-like* (29). Another drawback of the present study is the inability to perform sequencing of bacterial isolates to confirm the presence of resistance genes, due to the unavailability of sequencing platforms within the laboratory facility at the time of the study. As an alternative, the BioFire BCID2 panel was employed in cases of discrepancies for confirmatory purposes. This choice was based on several key considerations: BCID2 is widely used in clinical settings, it operates through multiplex PCR directly from positive BCs (similarly to the MM system) and, although it does not cover all the resistance genes included in the MM cartridges, it offers the highest overlap in terms of target coverage among the available reference platforms. This made BCID2 the most appropriate comparator for resolving discordant results in the absence of sequencing data. To further validate the MM system’s clinical utility, extended studies are recommended. For example, it is suggested to include fungal diagnostics, especially the MM YEAST BLOOD cartridge, to evaluate the platform’s comprehensive applicability in polymicrobial and fungemia cases. Also, large-scale clinical trials involving diverse real-world patient samples will help confirm the diagnostic accuracy across broader epidemiological settings, including low-prevalence pathogens and resistance genes. Likewise, comparative outcome studies, such as recently published ones (Mauri et al., 2024; Tićac et al., 2025), evaluating patient management, antibiotic use, and clinical outcomes when MM is integrated into routine care, contribute in quantifying its impact on sepsis treatment and healthcare efficiency. Software enhancement is also recommended to improve interpretation of low-level amplification signals and reduce false positives and negatives due to artifact-prone results. Although the tested assays are designed to provide results in approximately one hour, this study did not assess the impact of the reduced time to diagnosis on clinical outcomes. Nonetheless, the observed high diagnostic performance supports its potential utility as a valuable tool in clinical microbiology laboratories for the identification of bloodstream infection pathogens and antimicrobial resistance genes.

## Data Availability

The datasets presented in this study can be found in online repositories. The names of the repository/repositories and accession number(s) can be found in the article/**Supplementary Material**.
