# Supplementary material for: Evaluation of molecular mouse sepsis panel: new portable and rapid tests for microorganism detection in suspected blood stream infection
Source: Front Cell Infect Microbiol. 2025 Jul 29;15:1579074. doi: 10.3389/fcimb.2025.1579074 (PMC12339568; doi:10.3389/fcimb.2025.1579074)
Supplement: Supplementary file 1 [file DataSheet1.pdf]

S1: The preparation of 125 bacterial cultures (BCs) involved inoculating well-characterized strains or reference strains sourced from multiple recognized culture collections. The strains used are summarized in the table below, detailing their respective origin and culture collection identifiers.

STRAINS	MICROORGANISMS	RESISTANCE GENES
**ATCC 6633**	*B.subtilis*	
**ATCC 6305**	*S.pneumoniae*	
**ATCC 13813**	*S.agalactiae*	
**ATCC 19615**	*S.pyogenes*	
**ATCC 51299**	*E.faecalis*	
**ATCC 700221**	*E.faecium*	
**ATCC 19115**	*Listeria monocytogenes*	
**ATCC 33397**	*S.anginosus*	
**PEG 734-1-2**	*S. sciuri*	
**NCTC 13552**	*S.aureus* MRSA	mecC
**ATCC 27844**	*S.homins*	
**ATCC 49134**	*S.epidermidis*	
**ATCC 43300**	*S.aureus*	
**ATCC 49576**	*S.lugdunensis*	
**ATCC 29970**	*S.haemolyticus*	
**ATCC 29061**	*S.sciuri*	
**ATCC 27851**	*S.simulans*	
**ATCC 15305**	*S.saprophyticus*	
**ATCC 29971**	*S.xylosus*	
**ATCC 51299**	*E.faecalis*	vanB
**ATCC 700221**	*E.faecium*	vanA
**ATCC 700425**	*E.gallinarum*	vanC1
**NCTC 12361**	*E.casseliflavus*	vanC2-3
**PEG 954-1-1**	*S. sciuri*	mecA
**ATCC 35029**	*Klebsiella aerogenes*	
**NCTC 10102**	*Klebsiella aerogenes*	
**ATCC 13048**	*Klebsiella aerogenes*	
**ATCC 27853**	*Pseudomonas aeruginosa*	
**NCTC 10662**	*Pseudomonas aeruginosa*	
**ATCC BAA-1744**	*Pseudomonas aeruginosa*	
**ATCC 9344**	*H. influenzae*	
**NCTC 11931**	*H. influenzae*	
**PEG 16 2 5**	*H. influenzae*	
**ATCC 10211**	*H. influenzae*	
**ATCC 14028**	*Salmonella typhimurium*	
**ATCC 25931**	*Shigella sonnei*	
**ATCC 12022**	*Shigella flexneri*	
**ATCC BAA-2341**	*Enterobacter cloacae*	
**NCTC 13464**	*Enterobacter cloacae*	
**NCTC 13406**	*Enterobacter cloacae*	
**ATCC 49466**	*Acinetobacter baumannii*	
**ATCC 19606**	*Acinetobacter baumannii*	

**ATCC BAA-747**	*Acinetobacter baumannii*	
**NTCT-13301**	*Acinetobacter baumannii*	
**NCTC 13302**	*Acinetobacter baumannii*	
**NTCT 13303**	*Acinetobacter baumannii*	
**NCTC 13424**	*Acinetobacter baumannii*	
**NCTC 10257**	*Stenotrophomonas maltophilia*	
**ATCC 51331**	*Stenotrophomonas maltophilia*	
**ATCC 43861**	*Serratia marcescens*	
**ATCC 13090**	*Neisseria meningitidis*	
**ATCC 13077**	*Neisseria meningitidis*	
**ATCC 7002**	*Proteus mirabilis*	
**NCTC 10417**	*Klebsiella oxytoca*	
**ATCC 43863**	*Klebsiella oxytoca*	
**ATCC BAA-2146**	*Klebsiella pneumoniae*	
**ATCC BAA-2814**	*Klebsiella pneumoniae*	
**NCTC 13476**	*Escherichia coli*	
**CECT 409**	*Salmonella typhi*	
**NCTC 13476**	*Escherichia coli*	IMP
**ATCC 13462**	*Escherichia coli*	CTXM-2
**ATCC 13463**	*Escherichia coli*	CTXM-8
**ATCC BAA-199**	*Escherichia coli*	SHV ESBL
**CDC 1100192**	*Klebsiella pneumoniae*	NDM
**ATCC BAA-2471**	*Escherichia coli*	NDM
**ATCC BAA-2814**	*Klebsiella pneumoniae*	KPC TEM-1 SHV
**NCTC 13424**	*Acinetobacter baumannii*	OXA 23 like
**ATCC BAA-2341**	*Enterobacter cloacae*	KPC
**NCTC 13420**	*Acinetobacter baumannii*	OXA 51 like
**NCTC 13421**	*Acinetobacter baumannii*	OXA 23; OXA 51 like
**NCTC 13439**	*Klebsiella pneumoniae*	VIM
**NCTC 14337**	*Klebsiella pneumoniae*	IMP
**CCGU 55972**	*Escherichia coli*	CTXM-2
**NCTC 14320**	*Escherichia coli*	KPC IMP OXA-48
**NCTC 14321**	*Escherichia coli*	KPC OXA-48
**NCTC 14377**	*Escherichia coli*	mcr-1
**NCTC 13438**	*Klebsiella pneumoniae*	KPC
**NCTC 13440**	*Klebsiella pneumoniae*	VIM
**NCTC 14334**	*Klebsiella pneumoniae*	IMP
**NCTC 14322**	*Enterobacter cloacae*	KPC
**NCTC 14333**	*Escherichia coli*	NDM
**ATCC BAA-204**	*Escherichia coli*	SHV ESBL
**NCTC 14325**	*Escherichia coli*	NDM
**NCTC 14379**	*Escherichia coli*	mcr-3
**NCTC 13437**	*Pseudomonas aeruginosa*	VIM
**NCTC 14328**	*Enterobacter cloacae*	VIM
**CCUG 59343**	*Escherichia coli*	CMY-2
**NCTC 13452**	*Escherichia coli*	CTXM-3 TEM-1
**NCTC 13451**	*Escherichia coli*	CTXM-15 TEM-1

**NCTC 14378**	*Escherichia coli*	mcr-2
**DSM 109983**	*Pseudomonas aeruginosa*	TEM-1 SHV
**CCUG 62975**	*Escherichia coli*	CTXM-2
**NCTC 13450**	*Escherichia coli*	CTXM-15 TEM-1
**NCTC 14339**	*Escherichia coli*	NDM
**CCUG 59342**	*Escherichia coli*	CMY-2
**NCTC 14377**	*Escherichia coli*	mcr-1
**PEG 13-26-3**	*Escherichia coli*	SHV
**NCTC 13442**	*Klebsiella pneumoniae*	OXA-48
**CCUG 55972**	*Escherichia coli*	CTXM-2
**DSM 24599**	*Pseudomonas aeruginosa*	VIM
**NCTC 13353**	*Escherichia coli*	CTXM-15
**NCTC 13304**	*Acinetobacter baumannii*	OXA 27
**ATCC 700603**	*Klebsiella pneumoniae*	SHV ESBL
**NCTC 13846**	*Escherichia coli*	CTXM-27 TEM-1 mcr-1
**ATCC 13461**	*Escherichia coli*	CTXM-2
**ATCC 13464**	*Enterobacter cloacae*	CTXM-9
**PEG 13-56-37**	*Escherichia coli*	CTXM-27
**ATCC BAA-2341**	*Klebsiella pneumoniae*	KPC
**PEG 13-3-8**	*Escherichia coli*	CTXM-1
**PEG 13-26-70**	*Escherichia coli*	CTXM-1
**PEG 13-34-70**	*Escherichia coli*	CTXM-1 TEM-1
**PEG 13-55-81**	*Klebsiella oxytoca*	CTXM-1
**PEG 13-11-44**	*Escherichia coli*	CTXM-2 TEM-1
**PEG 13-95-12**	*Proteus mirabilis*	CTXM-9 TEM-1
**PEG 13-20-12**	*Escherichia coli*	CTXM-15
**PEG 13-38-24**	*Escherichia coli*	CTXM-55 CTXM-15
**PEG 13-62-64**	*Escherichia coli*	CTXM-15
**PEG 13-3-50**	*Klebsiella pneumoniae*	CTXM-15 TEM1 SHV
**PEG 13-3-53**	*Klebsiella pneumoniae*	SHV ESBL
**PEG 13-30-54**	*Klebsiella pneumoniae*	CTXM-3 TEM-1 SHV
**PEG 13-69-79**	*Klebsiella pneumoniae*	CTXM-9 SHV
**PEG 13-99-69**	*Klebsiella pneumoniae*	TEM-1 SHV
**PEG 13-55-75**	*Klebsiella pneumoniae*	CTXM-15 TEM-1 SHV
**ATCC BAA-2146**	*Klebsiella pneumoniae*	NDM CYM-2 CTXM-15 SHV
**NCTC 14339**	*Escherichia coli*	NDM

S2: Table of MM cartridge for the identification of gram-positive and gram-negative bacteria and antibiotic resistance genes

MM GRAM POS STAPH	MM GRAM POS NO STAPH
Staphylococcus aureus	*Enterococcus* spp.
Staphylococcus epidermidis	*Enterococcus faecalis*
Staphylococcus hominis	*Enterococcus faecium*
Staphylococcus spp.	*Streptococcus* spp.
Staphylococcus simulans	*Streptococcus agalactiae*
Staphylococcus haemolyticus	*Streptococcus anginosus*
Staphylococcus sciuri	*Streptococcus pneumoniae*
Staphylococcus xylosus	*Streptococcus pyogenes*
Staphylococcus lugdunensis	*Listeria monocytogenes*
Staphylococcus saprophyticus	*Bacillus subtilis*
mecA	vanA
mecC	vanB
SCCmec-OrfX	vanC1
vanA	vanC2/3
vanB	
**MM GRAM NEG ID**	**MM GRAM NEG RES**
Klebsiella oxytoca	NDM (Carba)
Klebsiella pneumoniae	KPC (Carba)
Klebsiella aerogenes	VIM (Carba)
Escherichia Coli/Shigella spp.	OXA-48-like (Carba)
Salmonella typhi	OXA-23-like (Carba)
Enterobacter cloacae	IMP (Carba)
Enterobacteriaceae	CTX-M-1/9 group (ESBL)
Pseudomonas aeruginosa	CTX-M-2/8 group (ESBL)
Haemophilus influenzae	SHV ESBL (ESBL)
Acinetobacter baumannii	SHV (β-lactamase, all)
Neisseria meningitidis	CMY2 (AmpC)
Stenotrophomonas maltophilia	mcr-1 (Colistin)
Serratia marcescens	mcr-2 (Colistin)
Proteus mirabilis	
Proteus spp.	

S3: Performance of the MM GRAM NEG ID cartridge (SI 1701.0102/L, Alifax Srl)
#: number of samples; %: calculated percentage of specificity or sensitivity; Confidence Interval (CI)

MM GRAM NEG ID Target	***Sensitivity***				***Specificity***			
	**#**	**%**	**95% CI**		**#**	**%**	**95% CI**	
S. typhi	21/21	**100**	84,54	100	506/506	**100**	99,25	100
N. meningitidis	5/5	**100**	56,55	100	523/523	**100**	99,27	100
S. maltophilia	12/12	**100**	75,75	100	516/516	**100**	99,26	100
H. influenzae	23/23	**100**	85,69	100	503/503	**100**	98,24	100
P. mirabilis	32/32	**100**	89,28	100	492/495	**99,4**	98,23	99,79
***Proteus* spp.**	36/38	**94,7**	82,71	98,54	490/490	**100**	99,22	100
K. oxytoca	22/22	**100**	85,13	100	506/506	**100**	99,25	100
K. pneumoniae	48/51	**94,1**	84,08	97,98	476/476	**100**	99,2	100
Enterobacteriaceae	266/270	**98,5**	96,25	99,42	257/257	**100**	98,53	100
A. baumannii	23/23	**100**	85,69	100	505/505	**100**	99,25	100

E. cloacae	34/36	**94,4**	81,86	98,46	488/488	**100**	99,22	100
***E. coli/Shigella* spp.**	114/114	**100**	96,74	100	413/414	**99,8**	98,64	99,96
S. marcescens	28/31	**90,3**	75,10	96,65	497/497	**100**	99,23	100
K. aerogenes	24/26	**92,3**	75,86	97,86	502/502	**100**	99,24	100
P. aeruginosa	43/46	**93,5**	82,50	97,76	482/482	**100**	99,21	100

S4: Performance of the MM GRAM NEG RES cartridge (SI 1701.0101/L, Alifax Srl)
#: number of samples; %: calculated percentage of specificity or sensitivity; Confidence Interval (CI)

MM GRAM NEG RES Target	***Sensitivity***				***Specificity***			
	**#**	**%**	**95% CI**		**#**	**%**	**95%CI**	
mcr-1	7/7	**100**	*64,57*	*100*	262/262	**100**	*98,55*	*100*
OXA-23-like	4/4	**100**	*51,01*	*100*	264/264	**100**	*98,57*	*100*
IMP	7/7	**100**	*64,57*	*100*	264/264	**100**	*98,57*	*100*
SHV	19/19	**100**	*83,18*	*100*	231/252	**91,7**	*87,60*	*94,49*
KPC	28/28	**100**	*87,94*	*100*	259/259	**100**	*98,54*	*100*
VIM	27/27	**100**	*87,54*	*100*	260/260	**100**	*98,54*	*100*
CMY-2	6/6	**100**	*60,97*	*100*	262/262	**100**	*98,55*	*100*
mcr-2	3/3	**100**	*43,85*	*100*	265/265	**100**	*98,57*	*100*
***CTX-M-2/8* group**	7/7	**100**	*64,57*	*100*	261/261	**100**	*98,55*	*100*
***OXA-48* like**	34/34	**100**	*89,85*	*100*	254/257	**98,8**	*96,62*	*99,60*
***CTX-M-1/9* group**	27/27	**100**	*87,54*	*100*	244/244	**100**	*98,45*	*100*
NDM	23/23	**100**	*85,69*	*100*	268/268	**100**	*98,59*	*100*
SHV ESBL	10/10	**100**	*72,25*	*100*	259/259	**100**	*98,54*	*100*

S5: Performance characteristics of the MM GRAM POS STAPH (SI 1701.0103/L, Alifax Srl)
#: number of samples; %: calculated percentage of specificity or sensitivity; Confidence Interval (CI)

MM GRAM POS STAPH Target	***Sensitivity***				***Specificity***			
	#	**%**	**95% CI**		#	**%**	**95% CI**	
S. hominis	20/20	**100**	*83,9*	*100*	313/314	**99,7**	*98,2*	*99,9*
***Staphylococcus* spp.**	159/169	**94,1**	*89,4*	*96,7*	165/165	**100**	*97,7*	*100*
S. epidermidis	33/34	**97,1**	*85,1*	*99,5*	299/299	**100**	*98,7*	*100*
vanB	5/5	**100**	*56,5*	*100*	292/292	**100**	*98,7*	*100*
vanA	5/5	**100**	*56,5*	*100*	292/292	**100**	*98,7*	*100*
S. haemolyticus	20/21	**95,2**	*77,3*	*99,1*	312/313	**99,7**	*98,2*	*99,9*
S. sciuri	7/7	**100**	*64,6*	*100*	326/326	**100**	*98,8*	*100*
S. simulans	10/10	**100**	*72,2*	*100*	324/324	**100**	*98,8*	*100*
S. aureus	49/50	**98,0**	*89,5*	*99,6*	284/284	**100**	*98,7*	*100*
mecA	77/77	**100**	*95,2*	*100*	253/256	**98,8**	*96,6*	*99,6*
mecC	9/9	**100**	*70,1*	*100*	324/324	**100**	*98,8*	*100*
S. lugdunensis	22/23	**95,8**	*79,0*	*99,2*	309/309	**100**	*98,8*	*100*
S. saprophyticus	6/6	**100**	*61.0*	*100*	328/328	**100**	*98,8*	*100*
S. xylosus	6/6	**100**	*61.0*	*100*	328/328	**100**	*98,8*	*100*
**SCC*mec-orfX***	21/24	**87,5**	*69,0*	*95,7*	308/308	**100**	*98,8*	*100*

S6: Performance characteristics of the MM GRAM POS NO STAPH (SI 1701.0104/L, Alifax Srl)
#: number of samples; %: calculated percentage of specificity or sensitivity; Confidence Interval (CI)

MM GRAM POS NO STAPH Target	***Sensitivity***				***Specificity***			
	#	**%**	**95% CI**		#	**%**	**95% CI**	
S. pyogenes	18/18	**100**	*82,4*	*100*	335/341	**98,2**	*96,2*	*99,2*
S. pneumoniae	20/20	**100**	*83,9*	*100*	339/339	**100**	*98,9*	*100*
S. agalactiae	19/19	**100**	*83,2*	*100*	340/340	**100**	*98,9*	*100*
van C1	1/1	**100**	*20,6*	*100*	326/326	**100**	*98,8*	*100*
vanB	9/9	**100**	*70,1*	*100*	317/318	**99,7**	*98,8*	*100*
vanA	9/9	**100**	*70,1*	*100*	318/318	**100**	*98,8*	*100*
***Enterococcus* spp.**	55/55	**100**	*93,5*	*100*	304/304	**100**	*98,7*	*100*
E. faecium	32/32	**100**	*89,3*	*100*	327/327	**100**	*98,8*	*100*
E. faecalis	23/23	**100**	*85,7*	*100*	336/336	**100**	*98,9*	*100*
van C2-3	1/1	**100**	*20,6*	*100*	325/325	**100**	*98,8*	*100*
***Streptococcus* spp.**	96/96	**100**	*96,1*	*100*	262/262	**100**	*98,5*	*100*
L. monocytogenes	18/18	**100**	*82,4*	*100*	341/341	**100**	*98,9*	*100*
B. subtilis	12/13	**92,3**	*66,7*	*98,6*	346/346	**100**	*98,9*	*100*
S. anginosus	20/20	**100**	*83,9*	*100*	338/339	**99,7**	*98,9*	*100*
